# Current Interventions to Prevent HTLV-1 Mother-to-Child Transmission and Their Effectiveness: A Systematic Review and Meta-Analysis

**DOI:** 10.3390/microorganisms10112227

**Published:** 2022-11-10

**Authors:** Carolina Rosadas, Graham P. Taylor

**Affiliations:** 1 Section of Virology, Department of Infectious Disease, Imperial College London, London W2 1PG, UK; 2 National Centre for Human Retrovirology, St Mary’s Hospital, Imperial College Healthcare NHS Trust, London W2 1NY, UK

**Keywords:** HTLV, mother-to-child transmission, prevention, public policies, breastfeeding, interventions, vertical transmission, virus, pregnancy

## Abstract

Human T lymphotropic virus 1 (HTLV-1) may be transmitted from mother to child and affects at least 5–10 million individuals worldwide, with severe consequences on health. Strategies to prevent transmission are important, as there is no treatment or vaccine. This systematic review aimed to identify interventions to prevent HTLV-1 mother-to-child transmission and to determine their effectiveness. Exclusive formula feeding, short-term breastfeeding, use of freeze–thaw milk, milk pasteurization, maternal and infant antiretroviral drugs, caesarean section, early clamping of umbilical cord, screening of milk donors and avoidance of cross-breastfeeding were identified as possible strategies. Avoidance of breastfeeding is an intervention that prevents 85% of transmissions. This strategy is recommended in Japan, Brazil, Colombia, Canada, Chile, Uruguay, the USA and some regions of French Guyana. Whilst breastfeeding for <3 months does not increase the risk of transmission compared to exclusive formula-feeding, concerns remain regarding the limited number of studies outside Japan, and the lack of information on women having higher risk of HTLV-1 transmission and on the ability of women to discontinue breastfeeding. Additional interventions are plausible, but data on their effectiveness are limited. The acceptance of interventions is high. These findings may guide healthcare professionals and support policymakers in implementing policies to avoid HTLV-1 mother-to-child transmission.

## 1. Introduction

Human T lymphotropic virus type 1 (HTLV-1) is a retrovirus that may be transmitted from seropositive mothers to their babies. It is estimated that 20% of babies breastfed from women living with HTLV-1 will become infected. Mother-to-child transmission (MTCT) also occurs in approximately 2.5–5% of exclusively formula-fed babies born from seropositive mothers, indicating that there are other transmission routes, although less common [1].

This neglected infection affects at least 5–10 million individuals worldwide and is associated with a range of diseases [2,3]. Recently, the World Health Organization (WHO) has shown renewed interest in HTLV-1 [1,4,5], and this virus has now been included in their strategic planning for sexually transmitted infections (STIs) [6]. HTLV-1 is the etiologic agent of adult T cell leukaemia/lymphoma (ATL), an aggressive neoplasm; HTLV-1 associated myelopathy (HAM), a chronic and progressive neurological disease; infective dermatitis; uveitis; and pulmonary disease, amongst other inflammatory conditions. New data on the impact of HTLV-1 on health have emerged, including an increase in all-cause mortality [3], increased risk of diabetes and chronic renal disease [7]. This reinforces the need for interventions to avoid new infections. This is particularly important as antiretroviral therapies are ineffective in established infection and as treatment of the complications is difficult. Currently, there is no vaccine to prevent HTLV infection.

There is a desire for evidence to support the implementation of policies to prevent HTLV-1 transmission [1,5,8]. The effectiveness of interventions to prevent HTLV-1 mother-to-child transmission was recently questioned by the UK national screening committee [9] and by the Peruvian Ministry of Health [10]. Here, a comprehensive, systematic review on the interventions available to prevent HTLV-1 mother-to-child transmission and their effectiveness is presented. From the perspective of healthcare professionals, this information is important to guide clinical practice. From the perspective of policy makers, these data are key for decision-making.

## 2. Materials and Methods

A systematic review of literature was performed focusing on interventions to prevent HTLV MTCT. The main objective was to assess the effectiveness of avoidance of breastfeeding. Secondary aims included the identification of alternative strategies to prevent HTLV MTCT and to verify the acceptance of such interventions.

For the primary outcome, a systematic review and meta-analysis was performed according to PRISMA guidelines. PubMed was searched using the combination of key terms for HTLV and mother-to-child transmission: “((HTLV) OR (Human t cell lymphotropic virus) OR (Human t lymphotropic virus) OR (human t leukemia virus) OR (human t cell leukemia virus)) AND ((mother-to-child transmission) OR (vertical transmission) OR (milk) OR (mother to child transmission))”. The search was not limited by year of publication or by type or language. Studies focusing exclusively on HTLV-2 and those without information about strategies to prevent transmission were excluded. 

Review Manager (RevMan) Version 5.4 (The Cochrane Collaboration, 2020) was used for the meta-analysis. Data on the prevalence of HTLV-1 infection in babies born from seropositive mothers who were exclusively formula-milk fed were compared to those who were breastfed. The risk ratio with 95% confidence interval was calculated using Mantel–Haenszel methods, considering random effects. A Forest plot was created. The quality of the studies was assessed using the Newcastle–Ottawa Scale. In this scoring system, the following categories are assessed: selection, comparability and outcome. In the selection criteria, studies are scored according to the representativeness of the exposed cohort, the selection of the non-exposed cohort, ascertainment of exposure and demonstration that the outcome of interest was not present at the start of the study. As most of the studies were based on anti-HTLV-1 antibody detection in the babies and antibodies are passively transferred during pregnancy and expected to be present in all babies born from seropositive mothers, this item was deemed as not applicable. Regarding comparability, studies scored one star if they considered the presence of HTLV-1-associated diseases in the mothers. History of blood transfusion was considered an additional factor to score on this category. Testing babies at least at 12 months was considered adequate.

Additional information on interventions to prevent mother-to-child transmission, such as strategy type, recommendation of policies, effectiveness of interventions and acceptance rates were extracted from the literature. Guidelines published by the Ministry of Health in English, French, Portuguese and Spanish speaking countries focusing on breastfeeding, maternal–child health and sexually transmitted infection were searched to identify policies and recommendations focusing on the prevention of HTLV-1 mother-to-child transmission (Chile, Brazil, Argentina, Uruguay, Paraguay, Jamaica, Trinidad and Tobago, Colombia, Peru, Venezuela, Bolivia, the USA, the UK, French Guyana, Barbados, South Africa, Canada and Australia).

## 3. Results and Discussion

### 3.1. Literature Search

**Figure 1:**
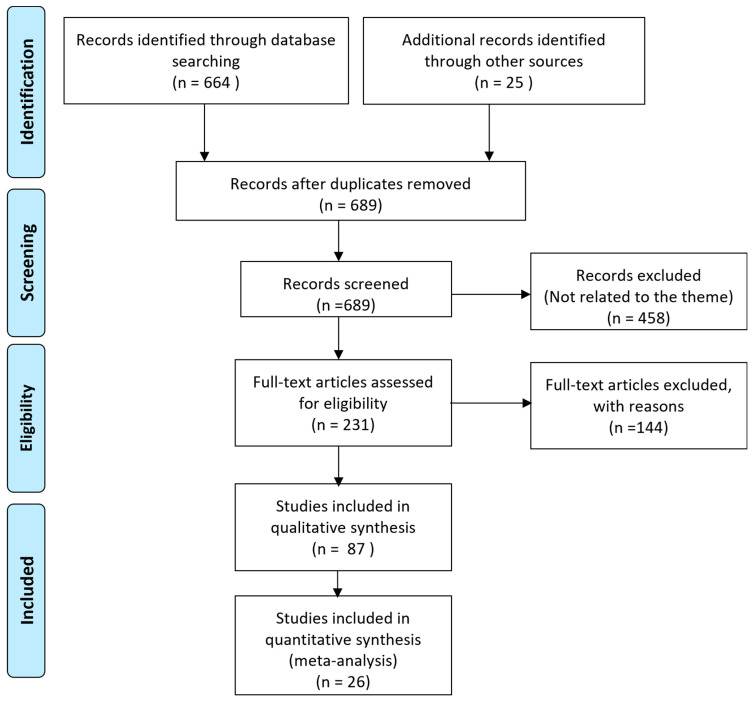
PRISMA diagram.

The literature search yielded 689 studies. After a first screen, 458 were excluded as they were duplicate or not related to the theme. After full-text reading, 87 studies were included (Figure 1).

### 3.2. Interventions Available to Prevent HTLV MTCT

The following interventions were identified in the literature: exclusive formula feeding, shortening the duration of breastfeeding, use of freeze and thaw milk [11,12,13,14,15,16,17], pasteurization of milk [18,19], use of milk bank [13], antiretroviral treatment (maternal and infant) [20,21,22], caesarean section [23], early clamping of umbilical cord [24], screening of milk donors [25,26] and avoidance of wet nursing. Multidisciplinary support for mothers living with HTLV-1 and training of healthcare professionals were also identified as important strategies for the effectiveness of any policy [27].

### 3.3. Effectiveness of Interventions

#### 3.3.1. Exclusive Formula Feeding

The first evidence that refraining from breastfeeding is effective in preventing HTLV-1 MTCT emerged in the 1980s. Hino et al. showed a marked difference in infection rates in bottle-fed babies (0%) compared to those who were breastfed (39%) [28]. This was confirmed by others. Following this remarkable discovery, an antenatal screening program commenced in Nagasaki, with the recommendation of exclusive bottle feeding for seropositive mothers. At that time, they estimated that the program would be able to prevent 85% of transmissions [29]. Looking back, Hino showed that the program was highly successful, with more than 90% of coverage of testing and acceptance of intervention, resulting in a reduction in transmission rates from 20 to 2.5%, which equates to 87% reduction, exceeding their first estimates. In addition, the prevalence of HTLV-1 in pregnant women in Nagasaki reduced from 7.1% in 1987, when the antenatal screening was implemented, to 1% twenty years later (2007), confirming the effectiveness of this policy [30].

**Figure 2:**
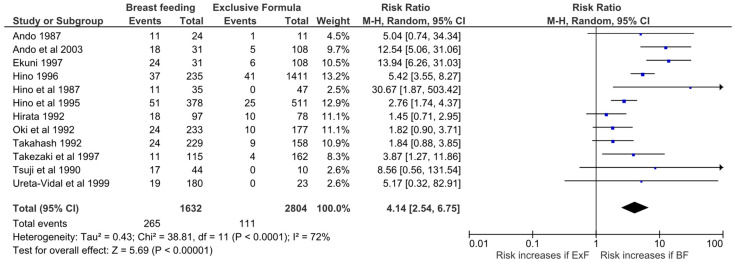
Forest Plot and meta-analysis showing the Risk Ratio of HTLV-1 infection in babies who were exclusive formula-fed and those who were breastfed by mothers living with HTLV-1. The boxes and lines indicate the risk ratios and their confidence intervals (CI) for each study. The pooled risk ratio is represented by a black diamond. The size of the blue squares indicates the relative weight of each estimate. Statistical analysis and graph were performed using RevMan 5 Software. Risk Ratio was calculated using Random effects model and Mantel-Haenszel statistical method [16,28,36,37,38,39,44,45,47,51,53,55].

**Figure 3:**
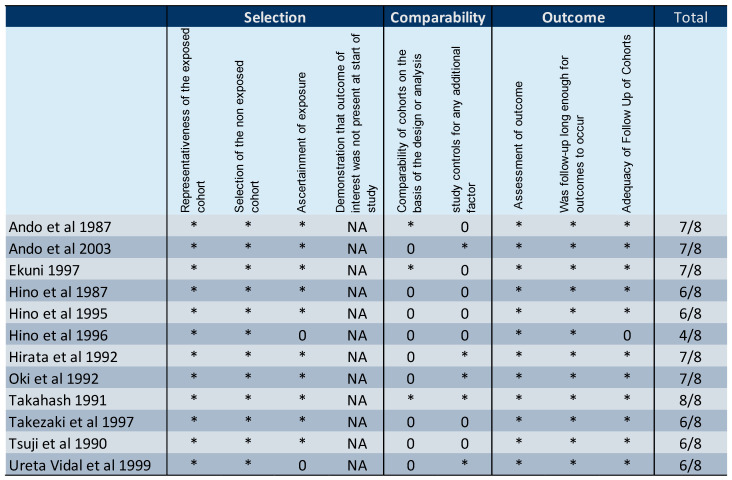
Quality assessment of the studies included in the meta-analysis by the Newcastle–Ottawa Scale. * represents one score [16,28,36,37,38,39,44,45,47,51,53,55].

The present systematic review on the effectiveness of refraining from breastfeeding included 26 studies [12,16,28,31,32,33,34,35,36,37,38,39,40,41,42,43,44,45,46,47,48,49,50,51]. Data on HTLV-1 transmission rates for those babies who were breastfed (regardless of the duration of breastfeeding) and those who were bottle fed were compared. There is a fourfold, statistically significant higher rate of infection among babies who are breastfed compared to exclusively formula-fed babies, confirming the effectiveness of refraining from breastfeeding to reduce HTLV-1 transmission to babies (Figure 2). The infection rate was 17.5% (414/2365) among breastfed infants versus 4.3% (124/2873) (Table 1) on those who received exclusively formula. This is similar to what was previously reported [52]. The quality assessment is shown on Figure 3. All studies, except for one, had a high score (≥6/8).

**Remark** **1.** *Avoidance of breastfeeding is a highly effective strategy preventing approximately 85% of HTLV-1 mother-to-child transmissions.*

Concerns have been raised about the recommendation of this policy in deprived areas [57,58]. It is important to note that formula feeding should be encouraged only when it meets the concept of AFASS, i.e., when exclusive formula is acceptable, feasible, affordable, sustainable and safe. The World Health Organization guideline updates for HIV and infant feeding points out the conditions needed to ensure safe formula feed [59]. These can be applied to those women living with HTLV-1 and are described below:

“(a) safe water and sanitation are assured at the household level and in the community; and

(b) the mother or other caregiver can reliably provide sufficient infant formula milk to support the normal growth and development of the infant; and

(c) the mother or caregiver can prepare it cleanly and frequently enough so that it is safe and carries a low risk of diarrhoea and malnutrition; and

(d) the mother or caregiver can exclusively give infant formula milk in the first six months; and

(e) the family is supportive of this practice; and

(f) the mother or caregiver can access health care that offers comprehensive child health services.”

[59]

**Remark** **2.** *Exclusive formula feeding should be encouraged for women living with HTLV-1 in settings where this intervention is acceptable, feasible, affordable, sustainable and safe.*

#### 3.3.2. Short-Term Breastfeeding

Recently, Itabashi and Miyazawa conducted a review of the effectiveness of short-term breastfeeding. The risk of HTLV-1 transmission to infants breastfed for 3 months or less was comparable to those who were fed exclusively with formula (relative risk (RR) (95% confidence interval (CI)): 0.72 (0.30–1.77)). However, when breastfeeding was carried out for up to 6 months, there was an almost threefold increase in risk (RR (95% CI): 2.91 (1.69–5.03)) [13]. 

There is a paucity of data on the risk of HTLV-1 transmission with short-term breastfeeding outside Japan, especially in women considered to have high risk of HTLV-1 transmission, such as those with HTLV-1 associated diseases, those co-infected by Strongyloides, those with high HTLV-1 proviral load and those who already have a child with an HTLV-1 infection [21,60].

**Remark** **3.** *Data indicate that breastfeeding up to 3 months does not increase the risk of HTLV-1 transmission compared to exclusive formula. However, individual risk factors should be considered before recommending this intervention. When exclusive formula-feeding does not meet AFASS criteria, short-term breastfeeding should be recommended.*

#### 3.3.3. Freeze–Thaw Milk

In the 1980s, Ando et al. showed that freezing milk at −20 °C overnight abolished HTLV-1 transmission in vitro [12]. The group then performed an intervention study and verified that all 13 children who received freeze–thaw milk from seropositive mothers were not infected at 12 months. They pointed out, however, that the duration of breast milk feeding was shorter than that with usual breastfeeding, but the exact duration was not presented [46]. Later, they observed that none of the 39 babies who received milk from seropositive mothers treated this way were infected after one year of follow up, and all 21 children who had long-term follow up remained negative. Again, the duration of human milk feeding was short, with an average of 2 months (Range: 2 weeks–6 months) [11]. In vitro data confirmed that the number of viable cells gradually decreases in milk according to the duration of storage at −20 °C (397 live cells/500 cells fresh milk, 324/500 3 h in the freezer, 15/500 after 6 h in the freezer and 0/500 when milk is frozen for 12 h) [16].

A recent meta-analysis showed that there is no difference in the risk of HTLV-1 transmission between babies who received freeze and thaw milk compared to those who were bottle-fed (risk ratio (95%CI): 1.14 (0.2–6.5)); however, the authors highlighted that any conclusions are hampered by the limited number of studies [13] and by the lack of comparison with babies who were breastfed for the same duration.

It is important to note that freezing and thawing milk may be considered time-consuming and a difficult strategy for new mothers, impacting the acceptance of this intervention. There is a limited number of studies addressing this intervention, and the duration of human milk feeding was short. The recommendation of this strategy is also limited in resource-limited areas, where there is no access to clean water, needed for sterilizing bottles, for example, or freezers.

**Remark** **4.** *Freezing and thawing milk may be effective to reduce transmission of HTLV-1 infection. However, the acceptance of this intervention is low and usually results in short-term duration.*

#### 3.3.4. Screening of Milk Donors, Pasteurization of Milk and Use of Milk Banks

Pasteurization of human milk has been proposed to prevent HTLV-1 MTCT [18], and some hypothesized that this would be effective [19] based on in vitro data showing that heat inactivates HTLV-1 [61]. However, there are no data available regarding the effectiveness of such a strategy. If effective, milk pasteurization could be interesting to avoid transmission via milk bank. Indeed, screening of milk donors for HTLV-1 is recommended in many areas. Although this policy is beneficial for identifying seropositive mothers, interventions to prevent infection to donor’s own infants will be limited. Mothers living with HTLV-1 will be informed and, therefore, be able to discontinue breast-feeding early, although not until they have already breastfed. 

There are no data regarding the use of milk from milk bank as opposed to formula for women who refrain from breastfeeding. The feasibility of this policy would be limited by the availability and access of milk bank by women living with HTLV-1. 

#### 3.3.5. Wet Nursing

In high-prevalence areas, where there is no HTLV-1 antenatal screening, cross-breastfeeding should be avoided as this would incur an additional risk of HTLV-1 transmission. It is thought that transplacental anti-HTLV-Ig protects the infants of infected mothers during the first 3 months. Wet nursed infants would have no such protection, and therefore, one cannot exclude a higher rate of transmission. Therefore, avoidance of cross-breastfeeding is preconized in some countries, such as Brazil. On the other hand, wet-nursing may be an alternative to formula for women who need to refrain from breastfeeding, as was recently proposed by the WHO for women infected by SARS-CoV-2 who are too unwell to breastfeed [62]. However, it is essential to screen wet nurses for HTLV-1 and other infections that may be transmitted via milk, such as HIV. In fact, HTLV-1 screening of wet nurses was proposed 30 years ago [63]. 

#### 3.3.6. Caesarean Section and Early Clamping of Umbilical Cord

There is no consensus on the effectiveness of caesarean section to prevent HTLV-1 infection. Immediate clamping of the umbilical cord is recommended in Brazil for mothers living with HTLV-1 [24]. Both recommendations aim to reduce the contact of neonate with maternal blood, assuming that this would decrease the risk of infection. Reduced contact with maternal blood was linked to reduced risk of transmission in Japan [64]. A study evaluated 41 children of seropositive mothers who were exclusively formula-fed, and no case of vertical transmission was detected. As 81.5% of them were delivered by c-section, the authors hypothesized that the delivery method may have contributed to the lack of transmissions in their cohort [50]. Recently, a management algorithm proposed that caesarean section should be considered for women with ATL and those with HTLV-1 proviral load in blood above 1% [21]. More data on the effectiveness of such interventions are needed, and one should balance the risks of those interventions, for both mother and child, particularly regarding caesarean section.

**Remark** **5.** *It is plausible that measures to reduce the contact of maternal blood with neonate would incur reduced risk of HTLV-1 perinatal transmission. More research is needed to clarify the benefits and risk of such interventions.*

#### 3.3.7. Antiretroviral Therapy

Leal and colleagues (2015) hypothesised that antiretroviral therapy would be effective in preventing perinatal HTLV-1 infection. They suggested conducting a trial in low- and middle-income countries, where infant morbidity and mortality is high [65]. The authors cite the effectiveness of Zidovudine in vitro against HTLV.

In a case series of four pregnant women with ATL in the UK, zidovudine was administered to all mothers during pregnancy and to the babies. All delivered by caesarean section and refrained from breastfeeding. Raltegravir was also administered to two mothers. One baby was infected, while the other three were HTLV-1 negative by PCR at 6 weeks, 3 months and 6 months [20]. It was proposed that antiretroviral therapy (zidovudine/Integrase strand transfer inhibitor, e.g., raltegravir) could be offered for women living with HTLV-1 and their babies if they have HTLV-1-associated diseases or are asymptomatic with proviral load higher than 1% in blood [21]. Although this is plausible, at the moment, there are no clinical data to support or refute this intervention. Novel data showed that Cabotegravir, the long-acting integrase strand transfer inhibitor, inhibits HTLV-1 transmission in vitro, raising the possibility of using this drug as pre-exposure prophylaxis [22].

**Remark** **6.** *Although the in vitro data are promising, there is a paucity of clinical data regarding the effectiveness of antiretroviral drugs to prevent HTLV-1 mother-to-child transmission.*

### 3.4. Policies Recommended Worldwide

In Nagasaki, HTLV-1/2 antenatal screening was implemented, and exclusive formula was recommended for seropositive mothers. Hino affirmed that experts did not feel confident to recommend short-term breastfeeding, based on available evidences [66]. Following the successful results observed in Nagasaki and due to patterns of migration in Japan, the country has opted to expand this policy. Therefore, in 2011, national HTLV antenatal screening was established in the country [67]. Initially, recommendations for pregnant women included exclusive bottle feeding, short-term breastfeeding, and use of freeze and thaw milk. According to Nishijima, this was recently revised (2016) and the current recommendation for seropositive mothers is exclusive formula feed [68].

In Brazil, cabergoline to help prevent the initiation of lactation along with the provision of formula milk has been recommended for seropositive mothers since 2019 [69,70]. The recommendation to screen pregnant women nationally was included in the recent national program for maternal and child health (2022), although some states had already implemented this policy. In Chile, antenatal screening and exclusive formula feeding is also recommended for seropositive mothers, except for babies with severe nutritional condition. In those cases, short-term breastfeeding (2–3 months) is recommended [71]. Since 1993, the Centers for Disease Control and Prevention (CDC) from the United States of America (USA) also advise women living with HTLV-1 to refrain from breastfeeding [72]. This is also recommended in Uruguay [73], Colombia [74], Canada [75] and some regions of French Guyana (Maripasoula, Papaïchton and Cayenne) [76]. In Argentina, HTLV-1 infection is considered a reason that may justify the suspension of breastfeeding [77]. In St Laurent du Maroni (French Guyana), women living with HTLV-1 are advised to opt for short-term breastfeeding (<3 months) [76].

**Remark** **7.** *Avoidance of breastfeeding is the recommended policy for women living with HTLV-1 in Japan, Brazil, Chile, Uruguay, Canada, Colombia, the USA and some regions of French Guyana. HTLV-1/2 antenatal screening is implemented in Japan and is recommended in Brazil and Chile.*

The National Institute for Health and Clinical Excellence from the UK (NICE) and the Human Milk Banking Association of North America (HMBANA) recommends screening of milk donors for HTLV-1 [78,79]. HTLV-1 screening of milk donors is recommended in France [19], Queensland Australia [80] and the UK and is carried out in 54% of milk banks in Europe [81]. In Sweden, according to the Swedish National Board of Health and Welfare (SNBHW), all donors should have a negative HTLV-1 test. However, a study revealed that only 52% of milk banks screen for HTLV-1 [82]. In a recent risk assessment on HTLV in human milk and human milk products, the Food Standards Australia and New Zealand, concluded that “ HTLV in imported human milk and human milk products presents a potential medium or high risk to public health and safety” [83].

**Remark** **8.** *Screening of milk donors for HTLV-1 infection is preconized by different institutions and implemented in many countries, demonstrating their concern about HTLV-1 transmission via milk.*

### 3.5. Women’s Choices: Acceptance of Interventions and Informed Decisions

In Nagasaki, the acceptance rate of bottle feeding was 80–90% [66]. In Kagoshima, Japan, acceptance of interventions was also extremely high (94.9%). In that region, most women (66.1%, 39/59) chose short-term breastfeeding (<3 months), while 17/59 (28.8%) seropositive women opted for bottle-feeding. None selected frozen–thawed milk and 3/59 could not chose their babies’ nutrition [84]. According to the authors, the recommendation differs from both settings. While in Kagoshima, women were advised to choose from short-term breastfeeding, exclusive formula feeding or freeze and thaw milk, in Nagasaki, they were advised to refrain from breastfeeding. This may, at least in part, explain the difference observed in the rates of women who chose exclusive bottle feeding in these two regions [84]. Another study compared the choices of pregnant women from Kagoshima and other regions from Japan and confirmed that although the acceptance of interventions for risk reduction is extremely high in both settings (>90%), the feeding-option selection differs between regions. The frequency of women who chose each method in Kagoshima and other regions in Japan were, respectively: long-term breastfeeding, 6/301 (5.6%) and 21/434 (4.8%); short-term breastfeeding (<3 months), 224/301 (74.4%) and 164/434 (37.8%); exclusive formula feeding, 70/301 (23.3%) and 213/434 (49.1%); and freeze and thaw milk, 1/301 (0.3%) and 36 (8.3%) [85].

**Remark** **9.** *Acceptance of interventions, specifically antenatal screening, and shortening or avoiding breast feeding, to prevent HTLV-1 mother-to-child transmission is high (above 90%).*

Information is a key component of any informed decision. Therefore, for women living with HTLV-1 to be able to make an informed decision, it is essential that both healthcare professional and women have sufficient and accurate information. However, this is not a reality in most cases. The lack of knowledge among healthcare professionals was identified as one of the main challenges faced by HTLV-1 individuals [5,8,86]. Many healthcare professionals are unaware about HTLV-1, including those working in endemic areas [86]. In Brazil, specialist advice on HTLV-1 helped mothers when they faced conflicting recommendations from healthcare professionals that were not aware about HTLV [87]. Therefore, training of healthcare professionals is a crucial component for the implementation of any policy.

Knowledge on HTLV-1 was limited, even among women who participated in an intervention study in Jamaica focused on the prevention of HTLV-1 mother-to-child transmission. In 2010, a minority was aware of HTLV-1 associated diseases, while 11.4% believed that HTLV-1 infection can cause HIV/AIDS. Only 33% knew that there was no cure for the virus. Only 58% knew that HTLV-1 is a sexually transmitted infection, and most women continued to have unprotected sex (88.6%) [88]. Therefore, increasing knowledge about HTLV-1 is essential to ensure that women are able to make an informed and conscient choice.

**Remark** **10.** *Training of healthcare workers is essential to guarantee that they can support women to make an informed decision regarding strategies to prevent HTLV-1 mother-to-transmission.*

Arguments on the psychological distress of HTLV-1 antenatal screening on women, as well as the impact of interventions on women’s wellbeing and health, have been raised. Evidence regarding the negative impact of such interventions is lacking. In addition, the impact that of the lack of interventions has on families in which transmissions have occurred is not documented. In Brazil, although women living with HTLV-1/2 may experience anxiety, sadness, fear and guilt [89] about refraining from breastfeeding, they also reported feeling relieved to be able to reduce the risk of HTLV-1 transmission to their babies [87].

### 3.6. Opportunity for Target Testing and Case Identification

As familiar aggregation is common in HTLV-1 infection [90,91,92,93], the identification of a women living with HTLV during antenatal screening becomes an opportunity for target testing of family members. This strategy was used in a study in Bahia, Brazil, which revealed that 32.5% of family members of women living with HTLV-1, identified during antenatal screening, were also seropositive for HTLV [94]. Contact tracing is a good strategy to optimise resources to identify new cases. This may be considered an added value of antenatal screening.

### 3.7. Integral Approach to Prevent Transmission

An integral approach is important to ensure that healthcare professionals are properly trained and that pregnant women have the support needed before screening and during notification of test results, and advice on strategies to reduce risk of transmission. A good practice was described in Bahia, Brazil, where a multidisciplinary centre organizes workshops for women living with HTLV-1 regarding strategies to increase the mother/child bond during bottle feeding and provides psychological support during and after pregnancy [27]. 

This integral support is important as many women have questions regarding preparation of formula and have difficulties following medical advice. In Kagoshima, 1/8 of women who choose short-term breastfeeding failed to stop at the recommended time. A trial with regular visits of public health nurse has started in the area [84], but the results have not yet been published. This difficult with stopping breast-feeding was confirmed in another study from Japan that revealed that up to 18% of the mothers who chose breastfeeding for 3 months continued breast-feeding for 4–6 months. Therefore, an integral strategy is needed to support those women to comply with the recommendation.

**Remark** **11.** *Mothers living with HTLV-1 may persist breastfeeding for longer than planned. Integral approach is essential to support those women living with this virus.*

## 4. Conclusions

Refraining from breastfeeding and provision of formula is the intervention with the largest body of evidence and is considered the most effective strategy currently available preventing 87% of early-life infections. This intervention has been recommended in the prefecture of Nagasaki since the establishment of antenatal screening, in the late 1980s. This is also recommended in Brazil, Chile, Uruguay, Canada, Colombia, the USA and some regions of French Guyana. When exclusive formula feed is not AFASS, short-term breastfeeding (up to 3 months) should be considered. Acceptance of HTLV-1 antenatal screening and interventions to limit or avoid breastfeeding is high (>90%). New interventions that will enable seropositive women to breastfeed their babies without imposing risk of HTLV-1 transmission are welcome, but in the meantime, there are interventions, such as exclusive formula-fed, that are highly effective to prevent HTLV-1 mother-to-child transmission and the implementation of such policies should be encouraged in all regions where HTLV-1 infections occur.

## Figures and Tables

**Table 1:** HTLV-1 transmission rates from mother to child according to feed strategy.

	Total Individuals Screened	HTLV-1 Positive	
		n	%
**Breast feeding**			
Ando et al. 1987 [45]	24	11	45.8
Ando et al., 1989 [46]	31	14	45.2
Ando et al., 2003 [47]	31	18	58.1
Barmpas et al., 2019 [48]	9	1	11.1
Biggar et al., 2006 [49]	162	28	17.3
Ekuni, 1997 [16]	31	24	77.4
Hino et al., 1987 [28]	35	11	31.4
Hino et al., 1995 [53]	378	51	13.5
Hino et al., 1996 [51]	235	37	15.7
Hirata et al., 1992 [44]	97	18	18.6
Houinato, 1998 [31]	86	20	23.3
Li et al., 2004 [33]	104	23	22.1
Mistro et al., 1994 [34]	9	2	22.2
Nakano et al., 1986 [54]	16	4	25.0
Nyambi, 1996 [35]	34	5	14.7
Oki et al., 1992 [36]	233	24	10.3
Takahash, 1991 [55]	229	24	10.5
Takezaki et al., 1997 [37]	115	15	13.0
Tsuji et al., 1990 [38]	44	17	38.6
Ureta Vidal et al., 1999 [39]	180	19	10.6
Van Tienen et al., 2012 [40]	51	13	25.5
Wiktor et al., 1993 [56]	34	5	14.7
Wiktor et al., 1997 [41]	181	28	15.5
Yoshinaga et al., 1995 [42]	16	6	37.5
**Total**	**2365**	**418**	**17.7**
**Exclusive formula feeding**			
Ando et al., 1987 [45]	11	1	9.1
Ando et al., 2003 [47]	108	5	4.6
Bittencourt et al., 2002 [50]	41	0	0.0
Ekuni, 1997 [16]	108	6	5.6
Hino et al., 1987 [28]	47	0	0.0
Hino et al., 1995 [53]	511	25	4.9
Hino et al., 1996 [51]	1141	41	3.6
Hirata et al., 1992 [44]	78	10	12.8
Kawase et al., 1992 [32]	298	13	4.4
Oki et al., 1992 [36]	177	10	5.6
Takahash et al., 1991 [55]	158	9	5.7
Takezaki et al., 1997 [37]	162	4	2.5
Tsuji et al., 1990 [38]	10	0	0.0
Ureta Vidal et al., 1999 [39]	23	0	0.0
**Total**	**2873**	**124**	**4.3**

## Data Availability

Not applicable.
